# Tailoring Magnetic Properties of Fe_0.65_Co_0.35_ Nanoparticles by Compositing with *RE*_2_O_3_ (*RE* = La, Nd, and Sm)

**DOI:** 10.3390/ma15207290

**Published:** 2022-10-18

**Authors:** Nacira Djellal, Paweł Pęczkowski, Djamel Eddine Mekki, Elena Navarro, Tarek Tahraoui, Jarosław Piętosa, Jan Marek Michalik, Pilar Marín, Łukasz Gondek

**Affiliations:** 1 Mines Metallurgy Materials Laboratory L3M, Department of Materials Science and Engineering, National Higher School of Mining and Metallurgy, Annaba 23000, Algeria; 2 Institute of Physical Sciences, Faculty of Mathematics and Natural Sciences, School of Exact Sciences, Cardinal Stefan Wyszyński University, K. Wóycickiego 1/3 Street, 01-938 Warsaw, Poland; 3 LESIMS, Department of Physics, Faculty of Science, University of Badji Mokhtar, Annaba 23000, Algeria; 4 Instituto de Magnetismo Aplicado, Universidad Complutense de Madrid (UCM-ADIF), 28230 Las Rozas, Spain; 5 Departamento de Física de Materiales, Universidad Complutense de Madrid (UCM), 28040 Madrid, Spain; 6 National Higher School of Technology and Engineering—ENSTI, Sidi Amar, Annaba 23000, Algeria; 7 Group of Phase Transition, Division of Physics of Magnetism, Institute of Physics, Polish Academy of Sciences, Lotników 32/46 Avenue, 02-668 Warsaw, Poland; 8 Department of Solid State Physics, Faculty of Physics and Applied Computer Science, AGH University of Science and Technology, A. Mickiewicza 30 Avenue, 30-059 Kraków, Poland

**Keywords:** Fe–Co, Fe_0.65_Co_0.35_, nanoparticles, rare-earth elements, soft magnetic materials

## Abstract

Fe-Co alloys are the most important soft magnetic materials, which are successfully used for a wide range of applications. In this work, the magnetic properties of lanthanide-substituted (Fe_0.65_Co_0.35_)_0.95_(*RE*_2_O_3_)_0.05_ (*RE* = La, Nd, and Sm) nanoparticles, prepared by mechanical alloying, are reported. Our comprehensive studies (X-ray diffraction, Mössbauer spectroscopy, scanning electron microscopy with X-ray energy dispersive spectrometry, SQUID magnetometry and differential scanning calorimetry) have revealed different properties, depending on the dopant type. The *RE*_2_O_3_ addition led to a decrease in the crystallite size and to an increase in the internal microstrain. Moreover, because of the high grain fragmentation tendency of *RE*_2_O_3_, the cold welding between Fe–Co ductile particles was minimized, indicating a significant decrease in the average particle size. The parent Fe_0.65_Co_0.35_ alloy is known for its soft ferromagnetism. For the La-substituted sample, the magnetic energy product was significantly lower (0.450 MG·Oe) than for the parent alloy (0.608 MG·Oe), and much higher for the Sm-substituted compound (0.710 MG·Oe). The processing route presented here, seems to be cost-effective for the large-scale production of soft magnetic materials.

## 1. Introduction

Soft magnetic nanoparticles (SMNs) represent an important field in materials science and engineering, since they exhibit unique and interesting characteristics that provide promising applications [1]. Typically, SMNs include ferrites, Fe–Ni, Fe–Si, Fe–Al, and Fe–Co based alloys, which have been studied intensively in recent years [1,2,3,4,5,6,7,8,9,10,11,12,13,14,15,16,17,18,19]. Fe–Co nanoparticles show high saturation magnetization and Curie temperature values, allowing the development of numerous applications, such as hyperthermia magnetic treatment [2] or thermoablative cancer therapy [3], magnetic resonance imaging (MRI) contrast [4,5] (currently the most commonly used gadolinium diethylenetriaminepentaacetic acid - Gd–DTPA [6]), high-density data storage [1,7], advanced materials for microwave devices [8], exchange-spring permanent magnets [1,9], and new generation of magnetorheological fluids [10]. Until now, it is generally known that the Fe_0.65_Co_0.35_ alloy has the highest saturation magnetization value. In order to obtain better magnetic properties, several substitutions on Fe_0.65_Co_0.35_, by various elements, have been undertaken, including Cr [11,12], Si [13,14], Si and Co [14] , Ni [15], Al [16], Cu [17], V [18], and Dy [19].

Another interesting observation is the combination of complementary features of Fe–Co (3*d* - itinerant magnetism) with rare-earth metals (4*f* – localized). The 4*f* rare-earth metals exhibit a strong magnetic susceptibility and high magnetocrystalline anisotropy, due to the interactions between their orbital moment and the crystalline field. Alloying them with a 3*d* metal induces their polarization, and therefore consolidates the magnetization of the alloy [20,21]. The rare-earth metals exhibit large ionic radii, which can modify the cell symmetry, and therefore generate internal stress, while substituting atoms with smaller ionic radii in the structure. Therefore, the structural, magnetic and magnetostrictive properties (e.g., cell parameter, average crystallite, and grain sizes) of the alloy are modified [21]. Studies on transition metal rare-earth (T-R) compounds show a fundamental interest in magnetic coupling and development of interface walls. Unfortunately, studies on T-R alloys are limited by the cost of rare-earth elements and their low oxidation stability. Interestingly, the Fe–Co system was not subjected, to the best of our knowledge, to compositing with rare-earth oxides. The motivation behind such research was the much lower cost of oxides when compared to pure elemental lanthanides.

In this work, we present the effects of *RE*_2_O_3_ (*RE* = La, Nd, and Sm) substitution on the structural, microstructural, morphological and magnetic behavior of mechanically alloyed Fe_0.65_Co_0.35_ nanoparticles. The mechanically alloyed Fe_0.65_Co_0.35_ compound exhibits the highest saturation magnetization within the Fe–Co family, and is well known for its soft magnetic properties [22,23]. 

## 2. Materials and Methods

### 2.1. Synthesis and Preparation of Samples 

Initial Fe (Alfa Aesar, 99%, *d* < 10 μm), Co (Alfa Aesar, 99.8%, 1.6 µm), La_2_O_3_ (Alfa Aesar, 99.9%), Nd_2_O_3_ (Alfa Aesar, 99.9%), and Sm_2_O_3_ (Alfa Aesar, 99.9%) powders were used to prepare the Fe_0.65_Co_0.35_ alloy and the corresponding (Fe_0.65_Co_0.35_)_0.95_(*RE*_2_O_3_)_0.05_ (*RE* = La, Nd, and Sm) samples with effective high-energy ball milling. It was found that 1% of impurity of Fe powder was caused mostly by oxygen (Fe_3_O_4_). The initial powders were mechanically alloyed (MA), in the appropriately prepared amounts, using a vibrating ball mill Retsch MM 400 with two cylindrical vials (25 mL, WC) and balls (10 mm, WC). The frequency of milling was kept at 20 Hz for 3 h, because for these mechanosynthesis parameters, the best structural and magnetic properties of the samples were obtained [22,23,24]. The ball to powder ratio was maintained at 25:1; around 50% of the vial volume was empty to assure suitable space for the milling process. In order to prevent excessive heating of the powders, the MA was stopped 15 min after every 15 min of milling.

### 2.2. Research Methods

The morphology and chemical composition of the samples were investigated with a JEOL-6100 scanning electron microscope (SEM), equipped with an X-ray energy dispersive spectrometer (EDS). The average particle size was estimated by scanning electron micrographs using ImageJ software.

The analyses of the structural properties were performed using the X-ray diffraction (XRD) method in an X’Pert MPD diffractometer. The anticathode of copper with *λ*_Kα1_ = 0.15406 nm was employed to obtain diffraction spectra. The range of 2*θ* was 5–100°, with a scanning step of 0.02°, and an exposure time of one second per step. The refined crystallite size, lattice parameter and microstrain were obtained using MAUD (Materials Analysis Using Diffraction) software.

In addition, ^57^Fe Mössbauer measurements were carried out at room temperature, in the transmission mode, utilizing a constant acceleration spectrometer with ^57^Co in a rhodium matrix as the source. The obtained spectra were fitted using the Gauss–Newton’s iterative method of minimizing the *χ*^2^, with a Lorentzian shape of the spectral lines. 

The measurements of the dependence of magnetization *M* as a function of the magnetic field *H* (*M*-*H* - hysteresis loops) were carried out using a superconducting quantum interference device (SQUID) magnetometer produced by Quantum Design GmbH in the applied magnetic field up to 50 kOe.

Structural phase transformations and magnetic ordering temperature were determined by differential scanning calorimetry (DSC), using DSC 404 NETZSCH. The measurements in the temperature range from 25 °C to 1200 °C were performed under protective nitrogen gas, with a heating rate of 30 °C/min.

## 3. Results and Discussion

### 3.1. Scanning Electron Microscopy (SEM) Analysis

**Figure 1:**
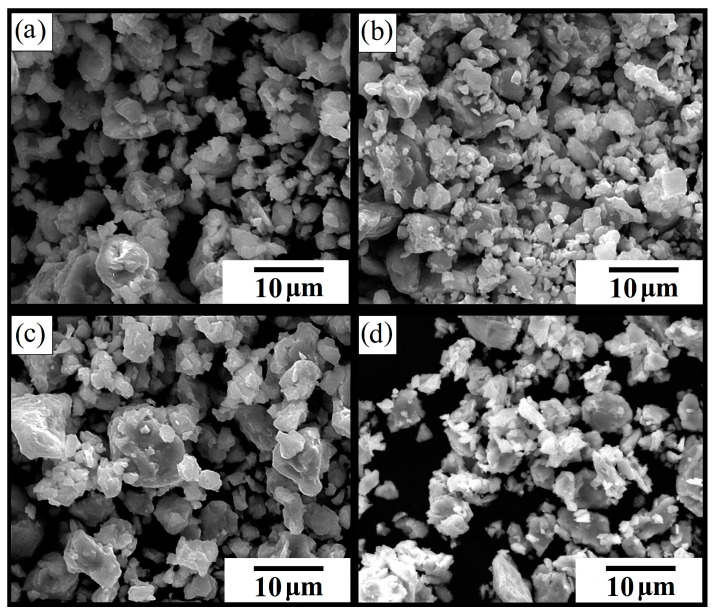
Scanning electron microscopy (SEM) images of the local microstructures of the following samples: (**a**) (Fe_0.65_Co_0.35_)_0.95_(La_2_O_3_)_0.05_, (**b**) (Fe_0.65_Co_0.35_)_0.95_(Nd_2_O_3_)_0.05_, (**c**) (Fe_0.65_Co_0.35_)_0.95_(Sm_2_O_3_)_0.05_, and (**d**) Fe_0.65_Co_0.35_ (3 h).

The morphology, particle size and chemical composition of pure and *RE*-substituted Fe_0.65_Co_0.35_ nanoparticles were investigated by SEM. Figure 1 shows the scanning electron microscopy (SEM) images of the local microstructures of the following samples: (Fe_0.65_Co_0.35_)_0.95_(La_2_O_3_)_0.05_ (**a**), (Fe_0.65_Co_0.35_)_0.95_(Nd_2_O_3_)_0.05_ (**b**), (Fe_0.65_Co_0.35_)_0.95_(Sm_2_O_3_)_0.05_ (**c**), and Fe_0.65_Co_0.35_ (3 h) (**d**).

**Figure 2:**
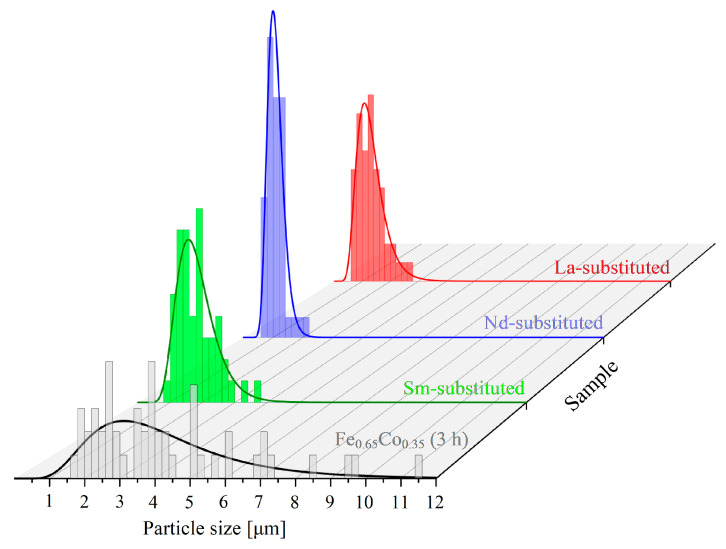
Average particle size distribution of Fe_0.65_Co_0.35_ and (Fe_0.65_Co_0.35_)_0.95_(*RE*_2_O_3_)_0.05_ (*RE* = La, Nd, and Sm) formed during effective high-energy ball milling for 3 h.

It can be observed that the particles of pure and *RE*-substituted Fe_0.65_Co_0.35_ (3 h) samples have an irregular shape. A large number of agglomerates and clusters was noticed. This was explained by the presence of strong magnetic interactions in the Fe–Co based alloys and by the high surface energy in the grain boundaries of powders produced during effective high-energy ball milling [25]. The average particle size distribution of all compositions is presented in Figure 2.

It can be observed that the average particle size distribution of *RE*-substituted samples is smaller than that for the pure Fe_0.65_Co_0.35_ (3 h) sample. This was explained by the increase in the hardness and brittleness of Fe-Co ductile powders after *RE*_2_O_3_ substitution [25].

The addition of *RE*_2_O_3_ leads to an increase in the grain fragmentation of Fe-Co powders, and to a decrease in the particle size. The decrement in the particle size after the *RE*-substitution can also be explained by the presence of secondary phases (NdFeO_3_ and LaFeO_3_) located at the grain boundaries, which can hinder the particles’ growth [26].

EDS analysis revealed elemental abundances, which are summarized in Table 1. It is thought that the samples show the appropriate stoichiometry. The short duration of high-energy milling prevented contamination from the milling vial and balls (tungsten carbide).

**Figure 3:**
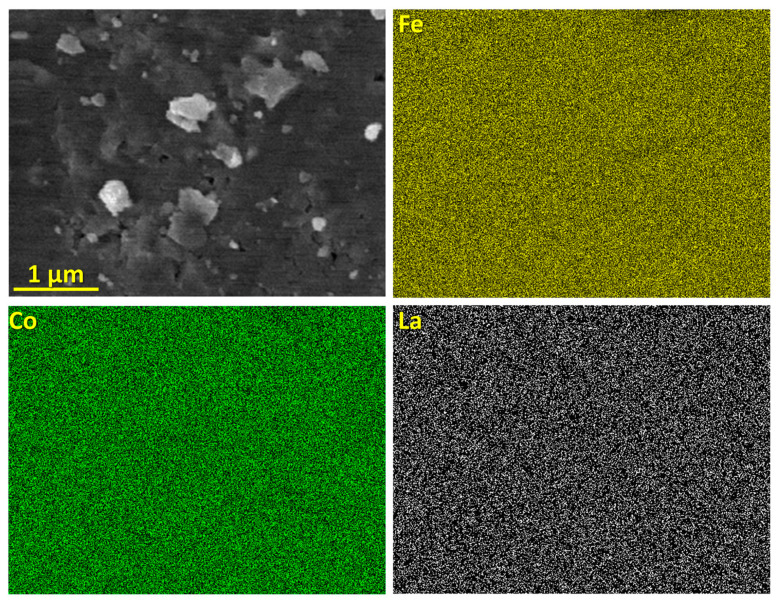
Chemical distribution of (Fe_0.65_Co_0.35_)_0.95_(La_2_O_3_)_0.05_ sample.

Figure 3 shows the chemical distribution of the (Fe_0.65_Co_0.35_)_0.95_(La_2_O_3_)_0.05_ sample. All constituents are homogenously dispersed in ferrite particles after 3 h of milling. This indicates that the elements are completely incorporated into the Fe structure. No traces of grains of *RE*_2_O_3_ oxides were detected.

### 3.2. X-ray Diffraction (XRD) Analysis

**Figure 4:**
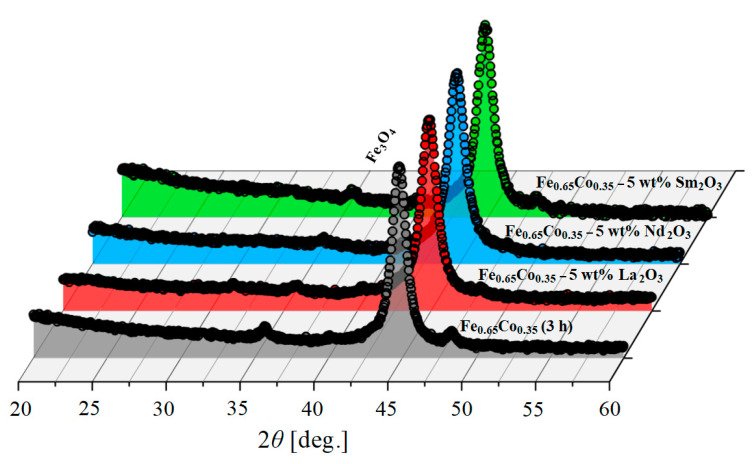
X-ray diffraction (XRD) patterns of Fe_0.65_Co_0.35_ (3 h) alloy and (Fe_0.65_Co_0.35_)_0.95_(*RE*_2_O_3_)_0.05_ samples with *RE* = La, Nd, and Sm. The Y-axis was square-rooted to magnify small peaks.

The structural and microstructural properties of the lanthanide-substituted Fe_0.65_Co_0.35_ (3 h) nanoparticles were investigated using the X-ray diffraction (XRD) technique. The XRD patterns of the prepared nanoparticles are shown in Figure 4.

**Figure 5:**
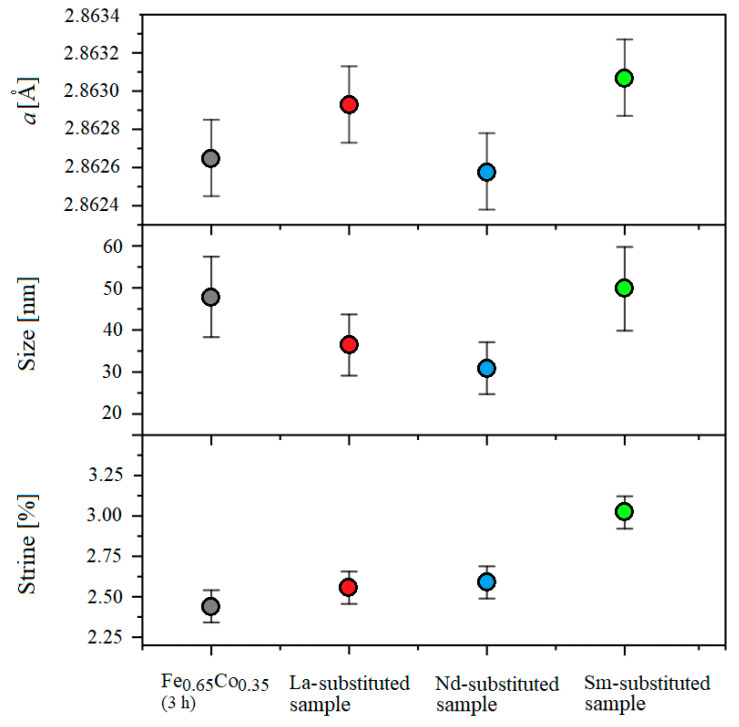
The crystallite size, microstrain and lattice parameter refined by the Rietveld analysis for pure Fe_0.65_Co_0.35_ alloy and for (Fe_0.65_Co_0.35_)_0.95_(*RE*_2_O_3_)_0.05_ samples with *RE* = La, Nd, and Sm.

Figure 4 shows the XRD spectrums of the Fe_0.65_Co_0.35_ (3 h) alloy and (Fe_0.65_Co_0.35_)_0.95_(*RE*_2_O_3_)_0.05_ samples with *RE* = La, Nd, and Sm. The characteristic peaks of Co, La_2_O_3_, Nd_2_O_3_, and Sm_2_O_3_ are no longer visible after 3 h of milling. The XRD patterns show the peaks characteristic for the body-centered cubic (bcc) iron structure (*Im*3*m*, COD 04-004-2474) for all investigated samples. These results confirm that Co, La^3+^, Nd^3+^, Sm^3+^, and O^2–^ were dissolved in the bcc-Fe structure. Traces of Fe_3_O_4_ are observed for all investigated specimens due to the initial impurity of Fe powder. Furthermore, extremely small peaks seem to originate from traces of the *RE*FeO_3_ phase, as presented by Suo et al. [27] and Kanna et al. [28]. The presence of these secondary phases suggests that the solubility of La^3+^ and Nd^3+^ inside the bcc-Fe structure is not complete due to their large radius (1.15 Å and 0.983 Å, respectively) [27]. However, the Sm^3+^ ions with a smaller radius are completely incorporated into the Fe_0.65_Co_0.35_ nanoparticles, as no additional peaks of any impurity phase were detected (Figure 4). The difference between the Fe and Co atomic radii is less than 15% and they also have the same valence (+3), which is necessary to reach a maximum solubility between atoms [29]. Moreover, the electro-negativity values of Fe and Co are almost the same, 1.83 and 1.88, respectively, which leads to a high solubility between them according to the Hume-Rothery rules [30,31]. The crystallite size, microstrain and lattice parameter refined by the Rietveld analysis, for the Fe_0.65_Co_0.35_ alloy and (Fe_0.65_Co_0.35_)_0.95_(*RE*_2_O_3_)_0.05_ samples with *RE* = La, Nd, and Sm, are shown in the Figure 5. The lattice parameters are the same for all the samples within the experimental error.

As shown in Figure 5, the average crystallite size in *RE*-substituted Fe_0.65_Co_0.35_ (3 h) samples is slightly smaller than that for the pure Fe_0.65_Co_0.35_ (3 h) alloy, except for the Sm-substituted sample. On the other hand, the microstrain values are larger in the (Fe_0.65_Co_0.35_)_0.95_(*RE*_2_O_3_)_0.05_ samples with *RE* = La, Nd, and Sm than in the Fe_0.65_Co_0.35_ alloy. This behavior could be explained by the difference between the mechanical alloying process of ductile–ductile powders (Fe–Co) and ductile–fragile ones (Fe, Co-*RE*_2_O_3_). In the first stage of ductile–fragile powders milling, the ductile particles (Fe, Co) exhibited plastic deformation, while the brittle particles (*RE*_2_O_3_) exhibited fragmentation. After the welding of the ductile particles, the fragile particles are placed between ductile particles at the collision time [32]. The fragmented particles are placed in the interfacial boundaries of the welded particles during effective high-energy ball milling. These successive phenomena, severe deformation, cold welding and solid dispersion, generate various defects (mainly dislocations) that lead to the increase in microstrain, material hardening and the enhancement of fragmentation. At the final stage, the equilibrium between the welding and fracture mechanisms is observed, leading to the formation of composite particles with a refined microstructure. In addition, the inclusions of the secondary phases at the grain boundaries inhibit the diffusion and hinder the growth of grains. The difference between Fe, Co metallic radii and *RE* covalent radii inside the lattice generates local disturbance and creates a strain in the crystal, which, in general, affects the nucleation rate and the crystallite sizes.

### 3.3. Mössbauer Spectrometry

**Figure 6:**
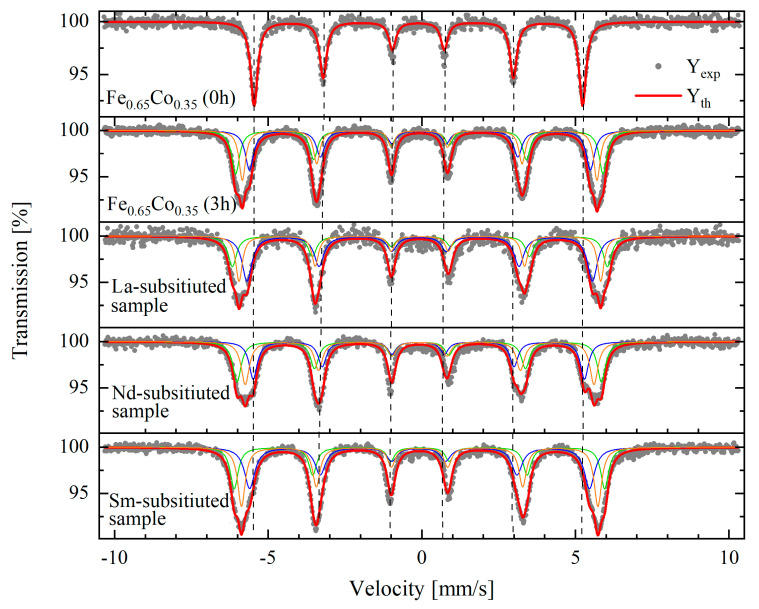
Mössbauer spectra of the base sample of Fe_0.65_Co_0.35_ (0 h), Fe_0.65_Co_0.35_ (3 h) alloy, and (Fe_0.65_Co_0.35_)_0.95_(*RE*_2_O_3_)_0.05_ samples with *RE* = La, Nd, and Sm milled for 3 h. Full circles - measured data; blue, orange and green - three fit components; red - resulting fit.

The Mössbauer spectra measured at room temperature for the base sample of Fe_0.65_Co_0.35_ (0 h) and Fe_0.65_Co_0.35_ (3 h) alloy, (Fe_0.65_Co_0.35_)_0.95_(*RE*_2_O_3_)_0.05_ samples with *RE* = La, Nd, and Sm milled for 3 h, are shown in the Figure 6, together with the calculated data. 

For the raw sample (mixture of initial powders), one sextet component was sufficient to satisfactorily fit the experimental data. The spectra for the Fe_0.65_Co_0.35_ alloy and composites with *RE*_2_O_3_ were fitted with three magnetically split (sextet) components. Hyperfine interaction parameters (isomer shift and hyperfine magnetic field) for each component (denoted as S1, S2, and S3), together with its relative contribution, are listed in the Table 2. In addition, the mean value of the hyperfine magnetic field, <*H*> and the isomer shift, with respect to *α*-Fe calibration <*IS*> for each sample, are presented in Table 2.

The Mössbauer spectrum of the raw Fe_0.65_Co_0.35_ (0 h) shows a sextet typical for magnetic behavior with a mean hyperfine magnetic field <*H*> ~ 33.0 T and an average isomer shift <*IS*> = 0.0021(10) mm/s. This behavior corresponds to the pure bcc-Fe, i.e., Fe has only Fe atoms in the neighbor’s shells, as no cobalt substitution takes place. This result shows that for a raw sample there is no Fe–Co interaction. After 3 h of milling, a broadening of the external lines is observed in the Fe_0.65_Co_0.35_ (3 h) Mössbauer spectra. This effect is attributed to the substitution of the Fe atoms by Co atoms in Fe–Co systems. The increase in the average hyperfine magnetic field to 35.7 T and the disappearance of the component with a magnetic field of 33.3 T is due to the formation of the Fe_0.65_Co_0.35_ (3 h) alloy, where Fe atoms coexist with randomly distributed Co atoms occupying bcc-Fe sites. This is further confirmed by an increase in the isomer shift up to 0.04 mm/s. These values are similar to those reported in previous works for the Fe–Co alloys [14,33].

The spectra of *RE*-substituted Fe_0.65_Co_0.35_ (3 h) samples show three sextets, indicating the coexistence of different magnetic environments of Fe atoms. The La-substituted Fe_0.65_Co_0.35_ (3 h) presents the highest average hyperfine field (36.1 T), which is in close agreement with the values found by Zelenakova et al. (36.16 T) [34]. For Nd- and Sm-substituted compositions, the average hyperfine field is about 35.0 T, which is in agreement with previous works for Fe–Co milled alloys [33,35,36]. The presence of *RE* elements in the Fe–Co structure may generate additional sextets in the Mössbauer spectra. The magnetic patterns were detected in the Mössbauer spectra of the *RE*-substituted Fe_0.65_Co_0.35_ (3 h) due to a small *RE* concentration.

### 3.4. The Magnetic Properties

**Figure 7:**
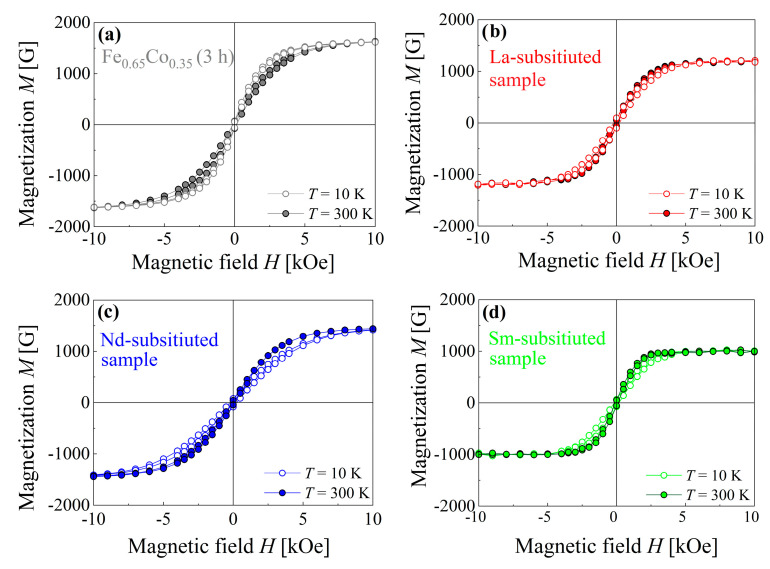
Magnetization *M* as a function of the magnetic field *H* for (**a**) Fe_0.65_Co_0.35_ (3 h) alloy and (Fe_0.65_Co_0.35_)_0.95_(*RE*_2_O_3_)_0.05_ samples with *RE* = (**b**) La, (**c**) Nd, and (**d**) Sm, recorded at 10 K and 300 K.

The effect of *RE*_2_O_3_ (*RE* = La, Nd, and Sm) addition on the magnetic properties of Fe_0.65_Co_0.35_ (3 h) nanoparticles was investigated, based on the *M* vs. *H* dependencies, recorded at 10 K and 300 K. Figure 7 presents the hysteresis loops for the Fe_0.65_Co_0.35_ (3 h) alloy and (Fe_0.65_Co_0.35_)_0.95_(*RE*_2_O_3_)_0.05_ samples with *RE* = La, Nd, and Sm showing soft ferromagnetic behavior.

Table 3 summarizes the magnetic properties of saturation magnetization (*M*_s_), coercive field (*H*_c_), remnant magnetization (*M*_r_) and magnetic energy product (*E_M_)* derived from these hysteresis loops for all investigated compositions.

For *T* = 300 K, the saturation magnetization value of Fe_0.65_Co_0.35_ (3 h) equals 1660(10) G, whereas the saturation magnetization values of the *RE*-substituted Fe_0.65_Co_0.35_ nanoparticles with La^3+^, Nd^3+^, and Sm^3+^ are equal to 1190(10) G, 1490(10) G, and 1000(10) G, respectively. The saturation magnetization value of the *RE*-substituted Fe_0.65_Co_0.35_ (3 h) nanoparticles decreased by 40% when compared with the pure one. The magnetic exchange interactions play a key role in the magnetization process in the nanoparticles. The *RE* (La^3+^, Nd^3+^, and Sm^3+^) ions partially replace Fe atoms. The ionic radii of La^3+^, Nd^3+^, and Sm^3+^ ions are higher than the atomic radii of Fe atoms, which results in the weakening of the exchange interactions. This causes a decrease in *M*_s_ for the substituted compounds; thus, the saturation magnetization of the Fe_0.65_Co_0.35_ (3 h) system is reduced [37,38]. 

The decrease in *M*_s_ for *RE*-substituted samples is caused by the decrease in the Fe–Fe and Fe–Co interactions (3*d*–3*d* coupling), due to the reduction in the concentration of Fe and Co ferromagnetic atoms, together with the presence of very weak *RE*–Fe (4*f*–3*d* coupling) and *RE*–*RE* (4*f*–4*f* coupling) interactions, when compared to 3*d*–3*d* ones [39,40]. The saturation magnetization value of *RE*-substituted Fe_0.65_Co_0.35_ nanoparticles depends mainly on their magnetic moments. The *M*_s_ value for Nd-substituted Fe_0.65_Co_0.35_ (3 h) nanoparticles is higher than for the other samples. This is explained by the fact that the Nd^3+^ ions have a higher magnetic moment (*J* = 3.6*µ*_B_), when compared with La^3+^ and Sm^3+^ ions (*J* = 0 and *J* = 1.38*µ*_B_) [41]. The coercivity values of *RE*-substituted Fe_0.65_Co_0.35_ (3 h) nanoparticles are higher than those for the pure Fe_0.65_Co_0.35_ nanoparticles. It is well known that the coercivity is strongly influenced by the microstructure and heavy plastic deformation during the MA process, which leads to the formation of defects and generation of internal strain inside the material [42,43]. 

The maximum coercivity values were reported for the La-, Nd-substituted Fe_0.65_Co_0.35_ (3 h) samples. As pointed out by XRD characterization, the solubility of La^3+^ and Nd^3+^ ions in the Fe_0.65_Co_0.35_ (3 h) structure is limited and some ions do not enter the Fe lattice structure, but precipitate as secondary phases at the grain boundaries. The antiferromagnetic behavior of these secondary phases significantly alters the magnetic response of the samples. Moreover, the presence of inclusions hinders the domain walls’ motion. As a result, the coercive field of the La- and Nd-substituted samples is higher (100(5) Oe for the La-substituted sample and 100(10) Oe for the Nd-substituted, respectively) than for other samples (85(5) Oe for the Sm-substituted sample and 75(5) Oe for the Fe_0.65_Co_0.35_ (3 h) alloy). However, for the La-substituted material, the magnetic energy product (*E*_M_) is the smallest (at 300 K), and is equal to 0.450 MG·Oe.

For *T* = 10 K, an enhancement of both *M*_s_ and *H*_c_ values of all prepared nanoparticles was noticed. The highest *M*_s_ value of 1515(15) G was observed for the Nd-substituted sample, which was ~2% higher than that observed at 300 K. In the La-substituted sample, we observed a lower value of saturation magnetization. The Sm-substituted nanoparticles demonstrate the lowest value of *M*_s_, 1025(10) G. The magnetization is mainly governed by the spin state and the magnetic moments of atoms; thus, the increase in *M*_s_ was principally due to the reduction in thermal fluctuation of the magnetic moments, and therefore the increase in magnetic ordering [44,45,46,47]. The coercivity has shown a substantial increase at 10 K, for all the investigated nanoparticles. For La- and Nd-substituted samples, the increase in *H*_c_ ranged from 100(5) Oe for 300 K to 290 K and 260(5) Oe for 10 K (by ∼66% and ∼62%, respectively). We noted a higher value for the Sm-substituted sample with an increase from 85(5) Oe for 300 K to 330(5) Oe for 10 K (by ~75%). It is apparent that *H*_c_ is strongly dependent on temperature. For a particle, thermal energy is essential to reverse its spin and to overcome the energy barrier. For *T* = 10 K, the particles did not have sufficient thermal energy; therefore, they required a stronger field to reverse the magnetization [48].

### 3.5. Differential Scanning Calorimetry (DSC) Analysis

**Figure 8:**
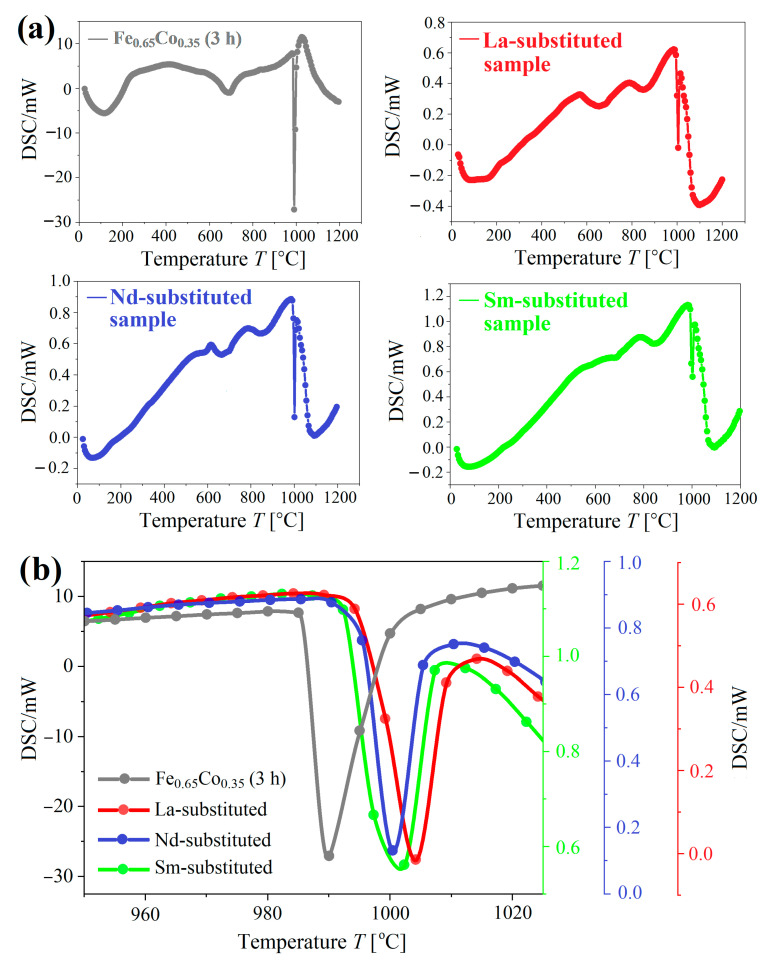
(**a**) The DSC curves for Fe_0.65_Co_0.35_ (3 h) alloy, and (Fe_0.65_Co_0.35_)_0.95_(*RE*_2_O_3_)_0.05_ samples with *RE* = La, Nd, and Sm, mechanically alloyed for 3 h in the mechanosynthesis process during effective high-energy ball milling. (**b**) The sharp peaks in all alloys related to the transition from body-centered cubic (bcc) ferromagnetic to the face-centered cubic (fcc) paramagnetic structure (range 950 – 1040 °C).

The structural stability was studied using the differential scanning calorimetry (DSC) method. Figure 8a presents the DSC curves of the Fe_0.65_Co_0.35_ (3 h) alloy, and (Fe_0.65_Co_0.35_)_0.95_(*RE*_2_O_3_)_0.05_ samples with *RE* = La, Nd, and Sm, mechanically alloyed for 3 h in the mechanosynthesis process during effective high-energy ball milling. The results confirm the formation of solid solutions.

A broad exothermic peak occurs at the temperature range 110 – 120 °C for all investigated compounds. This peak originates from the recovery, strain relaxation, grain growth and recrystallization of the nanocrystalline compositions [48]. The DSC scans show the presence of two main exothermic peaks. The first one is broad with the onset temperature of 670–680 °C, which can be attributed to the disordered (bcc) – ordered B2 (bcc) structural transformation. This is in good agreement with the Fe–Co phase diagram [49,50]. The second sharp peak observed for all investigated samples is related to the transition from body-centered cubic ferromagnetic to the face-centered cubic paramagnetic structure [50].

Note that the onset temperatures of the peaks are 987 °C, 994.8 °C, 995.6 °C, and 996.4 °C for Fe_0.65_Co_0.35_ (3 h) alloy, Sm-substituted sample – (Fe_0.65_Co_0.35_)_0.95_(Sm_2_O_3_)_0.05_, Nd-substituted sample – (Fe_0.65_Co_0.35_)_0.95_(Nd_2_O_3_)_0.05_, and La-substituted sample – (Fe_0.65_Co_0.35_)_0.95_(La_2_O_3_)_0.05_, respectively (Figure 8b). It seems that the *RE*_2_O_3_ addition stabilizes the bcc structure at high temperatures and increases the magnetic order temperature of the Fe_0.65_Co_0.35_ alloy.

## 4. Conclusions

The pure Fe_0.65_Co_0.35_ (3 h) alloy and (Fe_0.65_Co_0.35_)_0.95_(*RE*_2_O_3_)_0.05_ samples with *RE* = La, Nd, and Sm SMNs were successfully prepared with the mechanical alloying method. The research reported particles (agglomerates) of irregular shape that were 0.2 – 12 µm in size. According to EDS analysis, uniform distribution of the elements was achieved. The homogeneous phase formation in the investigated samples was confirmed using the XRD technique.

The X-ray diffraction patterns of the substituted Fe_0.65_Co_0.35_ (3 h) alloy demonstrated the bcc-Fe structure with traces of Fe_3_O_4_ that originated from the initial impurity of the Fe powder used. Rietveld refinement was used to obtain the lattice parameter, crystallite size and microstrain values. The *RE*-substituted Fe_0.65_Co_0.35_ nanoparticles showed a similar crystallite size (30–50 nm) and higher microstrain, when compared to the pure Fe_0.65_Co_0.35_ (3 h). The La-substituted sample seemed to behave differently from the other samples, presumably due to the larger La radius, compared to the other rare-earth metals. This was reflected in the hyperfine interactions, as it exhibited the largest mean isomeric shift and hyperfine magnetic field values (larger than for the parent alloy).

Magnetic measurements performed at 10 K and 300 K have shown the soft ferromagnetic nature of the (Fe_0.65_Co_0.35_)_0.95_(*RE*_2_O_3_)_0.05_ nanocomposites. The magnetization saturation and coercivity were found to be strongly dependent on *RE*-substitution and temperature. *RE*-substitution increased the magnitude of *H*_c_ and decreased the *M*_s_. At 300 K, the La-substituted sample was softer (0.450 MG^.^Oe) than the parent alloy (0.608 MG^.^Oe), requiring lower energy to reverse magnetization. On the other hand, for the Sm-substituted sample, higher energy was required to flip magnetization (0.710 MG^.^Oe). Substitution stabilizes the bcc structure at high temperatures, which is associated with an increase in the magnetic ordering temperature of the (Fe_0.65_Co_0.35_)_0.95_(*RE*_2_O_3_)_0.05_ samples, with respect to the parent alloy. For La-substituted sample, the highest ordering temperature of 1006 °C was reported. Low temperature behavior is also strongly modified by substitution with rare-earth metals. The coercive field increases at least by a factor of 2, while the remanence only slightly increases. The reported research shows a simple and effective route to produce novel materials with desired magnetic properties.

## Figures and Tables

**Table 1:** Results of X-ray microprobe analysis (EDS) recorded for the obtained powders.

Samples	Fe [at%]	Co [at%]	*RE* [at%]
Fe_0.65_Co_0.35_ (3 h)	65.2(1)	34.8(1)	-
Fe_0.65_Co_0.35_ – 5 wt% La_2_O_3_ (La-substituted sample)	64.3(1)	34.6(1)	1.1(1)
Fe_0.65_Co_0.35_ – 5 wt% Nd_2_O_3_ (Nd-substituted sample)	64.9(1)	34.0(1)	1.1(1)
Fe_0.65_Co_0.35_ – 5 wt% Sm_2_O_3_ (Sm-substituted sample)	64.4(1)	33.9(1)	1.2(1)

**Table 2:** Hyperfine interactions parameters for each sextet (*S*) (relative contribution and isomer shift with respect to Fe – *IS*; hyperfine magnetic field – *H*) for each component and their respective mean values (<*IS*>, <*H*>) obtained from the fits of measured Mössbauer spectra.

Samples	*S*	Rel. Contrib. [%]	*IS* Fe [mm/s]	*H* [T]	<*IS*> [mm/s]	<*H*> [T]
Fe_0.65_Co_0.35_ (0 h)	S1	100	0.002(1)	33.3(5)	---	---
Fe_0.65_Co_0.35_ (3 h)	S1	34.0	0.045(2)	37.0(4)	0.041	35.7
	S2	33.3	0.039(2)	34.3(4)		
	S3	32.7	0.039(3)	35.7(3)		
La-substituted sample	S1	25.7	0.068(7)	37.8(9)	0.048	36.1
	S2	44.3	0.034(5)	34.8(7)		
	S3	30.0	0.051(4)	36.4(5)		
Nd-substituted sample	S1	33.9	0.043(4)	36.7(5)	0.031	35.2
	S2	31.0	0.005(4)	33.4(4)		
	S3	35.1	0.040(4)	35.1(5)		
Sm-substituted sample	S1	27.2	0.045(3)	37.3(5)	0.037	35.7
	S2	35.6	0.025(4)	34.2(6)		
	S3	37.2	0.042(2)	35.9(3)		

**Table 3:** The magnetic properties of *RE*-substituted Fe_0.65_Co_0.35_ (3 h).

Compound	*M*_s_ [G]		*M*_r_ [G]		*H*_c_ [Oe]		*E*_M_ [MG^.^Oe]	
	10 [K]	300 [K]	10 [K]	300 [K]	10 [K]	300 [K]	10 [K]	300 [K]
Fe_0.65_Co_0.35_ (3 h)	1690(10)	1660(10)	67(7)	57(7)	130(2)	75(5)	1.414	0.649
La-substituted sample	1205(15)	1190(10)	97(7)	56(6)	290(5)	100(5)	0.630	0.450
Nd-substituted sample	1515(15)	1490(10)	78(8)	42(5)	260(5)	100(10)	3.312	0.608
Sm-substituted sample	1025(10)	1000(10)	68(8)	52(5)	330(5)	85(5)	1.688	0.710

## Data Availability

The data presented in this study are available on request from the corresponding author.
